# Identification and Characterization of the *AREB/ABF* Gene Family in Three Orchid Species and Functional Analysis of *DcaABI5* in *Arabidopsis*

**DOI:** 10.3390/plants13060774

**Published:** 2024-03-08

**Authors:** Xi Xie, Miaoyan Lin, Gengsheng Xiao, Qin Wang, Zhiyong Li

**Affiliations:** 1 Guangdong Provincial Key Laboratory of Lingnan Specialty Food Science and Technology, Zhongkai University of Agriculture and Engineering, Guangzhou 510225, China; xixie31@hotmail.com (X.X.); lin2040027358@163.com (M.L.); gshxiao@aliyun.com (G.X.); wangqin@zhku.edu.cn (Q.W.); 2 Key Laboratory of Molecular Design for Plant Cell Factory of Guangdong Higher Education Institutes, Department of Biology, Institute of Plant and Food Science, Southern University of Science and Technology, Shenzhen 518055, China; 3 Shenzhen Key Laboratory for Orchid Conservation and Utilization, The National Orchid Conservation Center of China and the Orchid Conservation & Research Center of Shenzhen, Shenzhen 518114, China

**Keywords:** orchid, gene family, *AREB*/*ABF* genes, *cis*-regulatory element (CRE) analysis, gene expression, abscisic acid (ABA), ABA INSENSITIVE 5 (ABI5)

## Abstract

*AREB*/*ABF* (ABA response element binding) proteins in plants are essential for stress responses, while our understanding of *AREB*/*ABFs* from orchid species, important traditional medicinal and ornamental plants, is limited. Here, twelve *AREB*/*ABF* genes were identified within three orchids’ complete genomes and classified into three groups through phylogenetic analysis, which was further supported with a combined analysis of their conserved motifs and gene structures. The *cis*-element analysis revealed that hormone response elements as well as light and stress response elements were widely rich in the *AREB/ABF*s. A prediction analysis of the orchid *ABRE*/*ABF*-mediated regulatory network was further constructed through *cis*-regulatory element (CRE) analysis of their promoter regions. And it revealed that several dominant transcriptional factor (TF) gene families were abundant as potential regulators of these orchid *AREB/ABF*s. Expression profile analysis using public transcriptomic data suggested that most *AREB/ABF* genes have distinct tissue-specific expression patterns in orchid plants. Additionally, DcaABI5 as a homolog of ABA INSENSITIVE 5 (ABI5) from *Arabidopsis* was selected for further analysis. The results showed that transgenic *Arabidopsis* overexpressing *DcaABI5* could rescue the ABA-insensitive phenotype in the mutant *abi5*. Collectively, these findings will provide valuable information on *AREB/ABF* genes in orchids.

## 1. Introduction

As one of the most species-rich plant families, the orchid family (Orchidaceae) consists of approximately 30,000 orchid species worldwide, which makes it a diverse and ecologically important plant family [1,2,3]. Beyond their ecological importance, orchids provide numerous sources to ecology, pharmaceuticals, food, and aesthetics [4,5,6,7]. Many orchid plants grow on shady mountain rocks or forest trunks, where they are often threatened by unfavorable environments such as drought and light exposure [8]. Hence, it is important to identify stress-related genes and investigate their functions in the orchid genome. In recent years, high-quality chromosome-scale assembly of genome sequences for several orchid species has been achieved [9,10,11,12]. Thus, valuable sequences in orchid genomes will provide genetic resources for the identification of important genes and valuable information for the further improvement of orchid varieties to increase stress resistance.

Under unfavorable growth conditions, plants will produce elevated levels of the stress-related hormone abscisic acid (ABA) [13]. ABA notably responds to stress damage by controlling the expression of abundant stress-related genes [14,15,16]. The ABA-mediated signaling pathway has been extensively studied in the model plant *Arabidopsis*. Generally, ABA binds to the receptor PYR/PYL/RCAR and inhibits the protein phosphatase activity of PP2Cs, which hinder the kinase activity of sucrose non-fermenting 2-related protein kinases (SnRK2s). Subsequently, the accumulation of the activated SnRK2 kinases directly targets downstream ABA-responsive genes, including the ABA-responsive element binding protein/ABRE-binding factor (AREB/ABF) subfamily members, allowing activation of the expression of target genes [17,18,19,20,21]. It has been also proposed that the ABA-independent pathway participates with particularly important transcriptional factors (TFs) in stress response. For instance, DEHYDRATION-RESPONSIVE ELEMENT-BINDING PROTEIN2A (DREB2A) is responsible for osmotic-stress-responsive gene expression in the ABA-independent pathway [22].

The AREB/ABF subfamily, which belongs to a subgroup of the basic leucine zipper (bZIP) TF family, consists of the most essential representatives in the ABA signaling pathway and plays important roles in plant responses to stresses [23,24,25]. In *Arabidopsis*, nine members (ABF1, ABF2, ABF3, ABF4, AtABI5, AtDPBF2, AtDPBF3, AtDPBF4, and AtbZIP15) constitute the AREB/ABF subfamily, which is classified into group A of the bZIP family with ten groups [2]. As one of the largest TF families, the bZIP gene family is the most abundant and evolutionarily conserved gene family in plants. It has been determined that the highly conserved bZIP domain mostly binds to the *cis*-elements using an ACGT core motif of target genes’ promoters, including TACGTA (A-box), GACGTC (C-box), and CACGTG (G-box) [26,27]. Numerous ABA and/or stress-regulated genes contain a (C/T)ACGTGGC consensus sequence, known as the ABA-responsive element (ABRE), in their promoter regions, which is directly targeted by *AREB*/*ABF* genes, including *AREB1/ABF2* and *ABF3*, under stressful conditions [28,29,30,31,32]. Many studies have demonstrated that the regulatory effects of AREB/ABFs on their target genes are mainly induced by stresses and thereby contribute to stress tolerance in plants [33,34]. For instance, plants overexpressing AREB2/ABF4 or ABF3 show increased sensitivity to ABA, whereas reduced expression of ABA/stress-regulated genes exhibited enhanced drought tolerance [33]. ABI5 is well known as the core transcriptional factor in the ABA signaling pathway, which could contribute to various types of stress tolerance, including drought, salt, cold, and heat [35,36].

Previous studies have reported *AREB/ABF* genes from various plants improve stress adaptability and resistance, such as in *Arabidopsis* [24], rice [34], wheat [37,38,39], potato [40,41], cotton [42], apple [43], strawberry [44], rose [45], lily [46], kiwifruit [47], and jute [48]. To date, the AREB/ABF subfamily in orchid plants remains largely unknown. In the present study, twelve *AREB/ABF* genes were identified within three orchids’ complete genomes: *Dendrobium catenatum* (*D. catenatum*), *Apostasia shenzhenica* (*A. shenzhenica*), and *Phalaenopsis equestri* (*P. equestris*). The features of the *AREB/ABF* gene family were characterized using combined bioinformatics methods. And the representative *AREB/ABF* member *DcaABI5* from *D. catenatum* was demonstrated to play an essential role in the ABA signaling response. Collectively, our results will provide valuable information on *AREB/ABF* genes and provide a perspective for further functional characterization of potentially important ones in orchids.

## 2. Results

### 2.1. Identification and Characteristics of Orchid AREB/ABFs

The AREB/ABF subfamily members from the three orchid species (*D. catenatum*, *A. shenzhenica*, and *P. equestris*) were identified using AREB/ABF proteins from *Arabidopsis* as the queries to search for candidate genes. After strict screening and sequence analysis, 5, 4, and 3 *AREB/ABF* genes were identified from *D. catenatum*, *A. shenzhenica*, and *P. equestris*, respectively. These *AREB/ABF* genes were further named according to the similarity to the homologs from *Arabidopsis*. Sequence analysis revealed that the coding sequences of these *AREB/ABF* genes in orchids ranged from 1278 bp (*AshABF2*) to 936 bp (*DcaDPBF2*) with an average of 1146 bp (Table 1). Analysis using ExPasy (https://www.expasy.org, accessed on 10 July 2021) showed that the molecular mass of them ranged from 34.83 kDa (DcaABI5) to 45.56 kDa (AshABF2), and their isoelectric points (pI) varied widely, from 5.47 (AshABI5) to 9.5 (DcaDPBF2). Additionally, the predicted subcellular localizations of these proteins suggested that all AREB/ABF subfamily proteins may be located in the nucleus.

**Figure 1:**
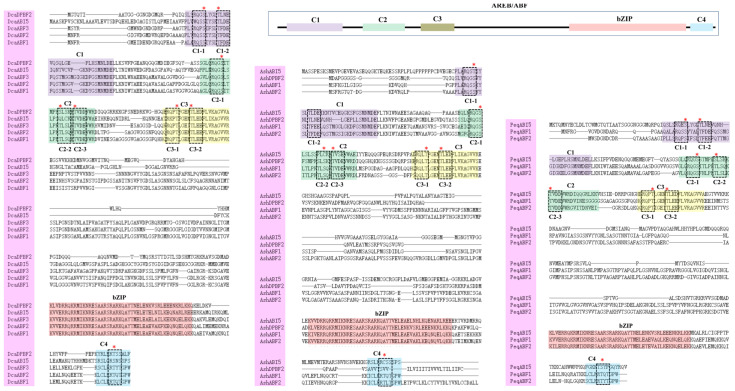
Multiple-sequence alignment of AREB/ABF members from *D. catenatum*, *A. shenzhenica*, and *P. equestris*. The positions of C1 to C4 conserved domains and basic bZIP regions are represented with different colors. Potential phosphorylated residues (R-S-SX/T) of the characteristic phosphorylation sites are indicated with dash boxes and red stars.

Typical AREB/ABFs have a highly conserved protein structure, including conserved phosphorylation domains (C1 to C4) with kinase recognition motifs (RXXS/T or S/TXXE/D) and a bZIP domain [49]. Multiple-sequence alignment analysis revealed that AREB/ABF proteins from the three orchid species contained all these conserved domains and kinase recognition motifs, which provides further support for their identities (Figure 1).

### 2.2. Phylogenetic Analysis of Orchid AREB/ABF Proteins

**Figure 2:**
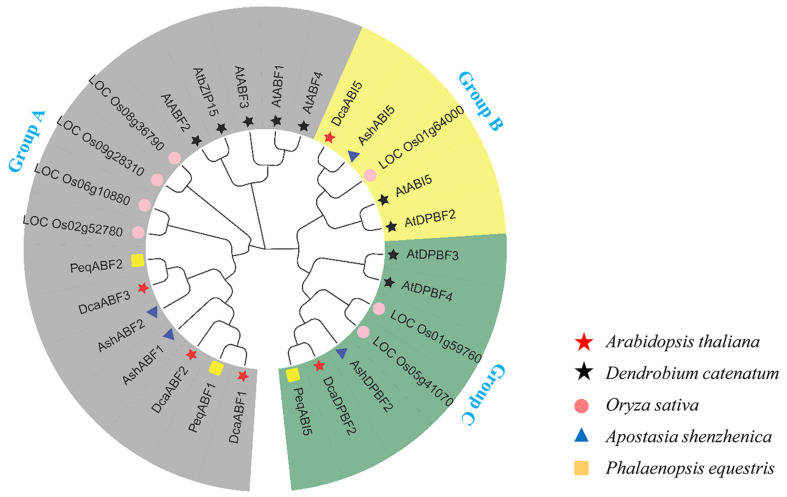
Phylogenetic analysis of the AREB/ABF proteins. The diverse groups of the AREB/ABF proteins are indicated with different colored arcs. Proteins from *D. catenatum*, *A. shenzhenica*, *P. equestris*, rice (*Oryza sativa*), and *Arabidopsis* are indicated using black stars, blue triangle, yellow squares, purple circles, and red stars, respectively.

To investigate the classification and evolutionary characteristics of the *AREB/ABF* proteins from *D. catenatum*, *A. shenzhenica*, and *P. equestris*, a non-rooted phylogenetic tree was constructed according to the multiple-sequence alignments of the orchid AREB/ABF proteins with their homologs from *Arabidopsis thaliana* (9) and *Oryza sativa* (7). As a result, the phylogenetic tree showed that the these AREB/ABF proteins were mainly divided into three groups (Figure 2). All the ABF proteins were clustered in Group A, and the ABF proteins from the orchid species exhibited a close relationship. However, the ABF proteins from *Arabidopsis* and rice were individually clustered apart from the homologs from the orchids. The ABI5 and DPBF proteins were clustered into two other groups and distributed evenly among all species.

### 2.3. Conserved Motifs and Gene Structure Analysis of the Orchid AREB/ABF Genes

**Figure 3:**
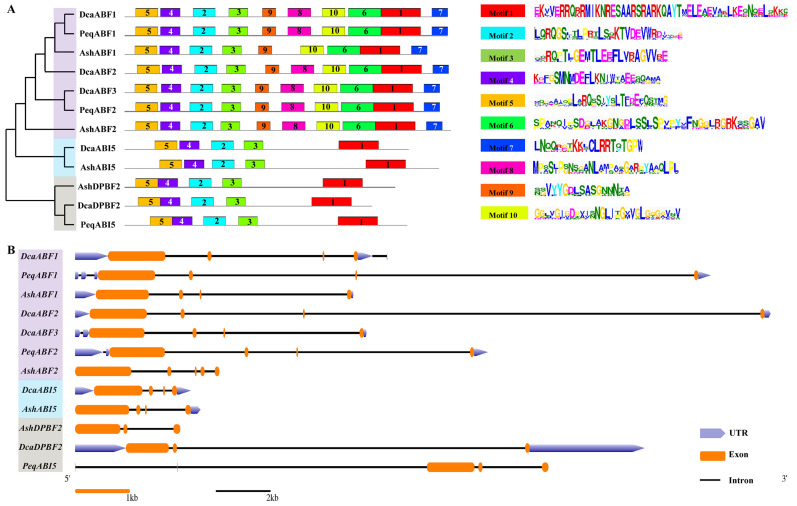
Architecture of conserved motifs and gene structures of *AREB/ABF*s. (**A**) The neighbor-joining phylogenetic tree was produced using MEGA using the neighbor-joining method with 1000 bootstrap replicates. Schematic represents the conserved motifs of the AREB/ABFs identified using MEME. Each motif is indicated by a colored box, number, and sequence. (**B**) Intron/exon structures of *AREB/ABF* genes. Exon(s), intron(s), and UTR(s) are represented with yellow boxes, black lines, and blue arrows, respectively.

To explore the conservation and diversification of the orchid AREB/ABFs, their putative motifs were predicted using the MEME Suite (https://meme-suite.org/meme/tools/meme, accessed on 25 July 2021) according to full-length phylogenetic relationships. The result showed that a total of 10 motifs ranging from 17 to 49 amino acids were identified from all the orchid AREB/ABFs and further labeled in order as the corresponding motifs 1–10. For individual AREB/ABF proteins, the number of conserved motifs ranged from 5 to 10, and those AREB/ABFs in the same group or subgroup comprised highly similar motif compositions (Figure 3A). Additionally, all the AREB/ABFs from group I in the phylogenetic analysis had 10 motifs except for AshABF1, which was short of motif 8. The AREB/ABFs from the other two groups contained only five motifs. Notably, AREB/ABF from group C had no gap between motif 4 and motif 5.

To further detect the structural features and evolutionary events of these orchid *AREB/ABF* genes, their exon/intron distributions were analyzed. The results showed that the *AREB/ABF* genes from group I generally had four exons and long introns except for AsABF2, which contained five exons and short introns (Figure 3B). The *AREB/ABF* genes from group II also had four exons but short introns. Additionally, the *AREB/ABF* genes from group III possessed either three or five exons and variable-length introns. 

Collectively, the motif composition and exon/intron distributions of *AREB/ABF* genes from the same group were closer than genes from different groups.

### 2.4. Cis-Element Analysis of the Orchid AREB/ABF Family

**Figure 4:**
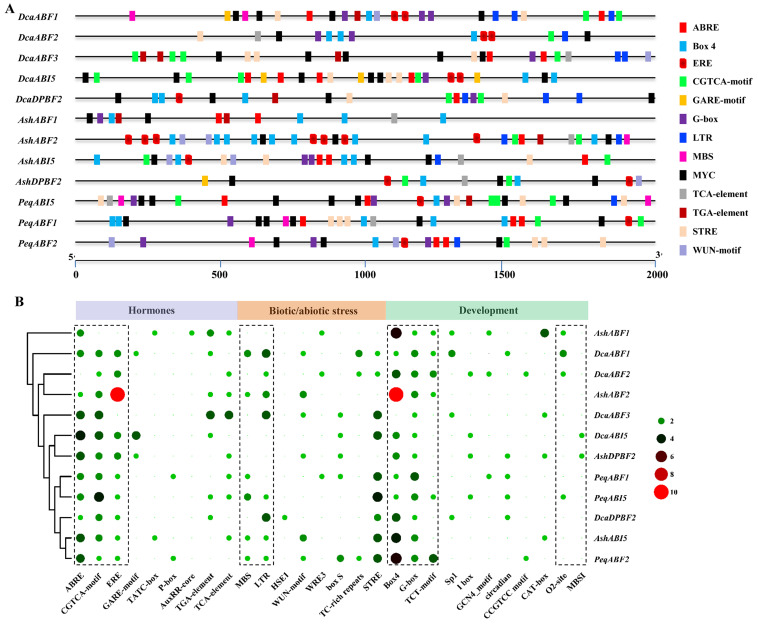
Analysis of *cis*-elements in the promoter regions of *AREB/ABF* genes from orchids. (**A**) The distribution of *cis*-elements to each *AREB/ABF* gene. Different colored blocks represent the corresponding *cis*-elements. (**B**) Evaluation of *cis*-elements of each *AREB/ABF* gene. The number of individual elements is indicated with a colorful circle.

To explore the putative *cis*-elements in the *AREB/ABF* genes, the 2000 bp upstream sequences of the *AREB/ABF* genes were investigated. As predicted, the composition of many *cis*-acting elements was detected (Figure 4A). For instance, the phytohormone-responsive elements mainly correlated with ABA (ABRE), ethylene (ERE), GA (GARE-motif, P box, TATC-box), JA (CGTCA-motif), auxin (AuxRR core, TGA-element), and SA (TCA-element) were widely predicted in the promoter regions of the orchid *AREB/ABF*s. Among them, ABRE, ERE, and CGTCA-motif were most enriched in all the *AREB/ABF* genes. Defense and stress response elements were also widely found within these *AREB/ABF* genes, including MBS (drought response element), LTR (low-temperature-responsive element), WUN-motif, and WRE3 (wound-responsive element). Notably, the dominant stress response element STRE (stress-responsive element) was presented in most of the *AREB/ABF* genes, except for *AsABF1*, *AsABF2*, and *AsDEPB2*. The most enriched *cis*-elements found were related to light response, including Box 4, G-box, GATA-motif, I-box, and TCT-motif. Additionally, some elements associated with tissue-specific expression were also detected. For instance, three CAT-box elements related to meristem expression were found in the promoter region of *AsABF1*. A circadian element related to circadian expression patterns was found in some of the *AREB/ABF* genes. Moreover, two other lesser-known elements were detected, including O2-site (zein metabolism regulation) and MBSI (flavonoid biosynthetic genes regulation). These results provide fundamental clues regarding the possible functions and expression patterns of *AREB/ABF* genes related to the composition of their *cis*-acting elements.

### 2.5. Prediction Analysis of the AREB/ABF-Mediated Regulatory Network in Orchid Plants

**Figure 5:**
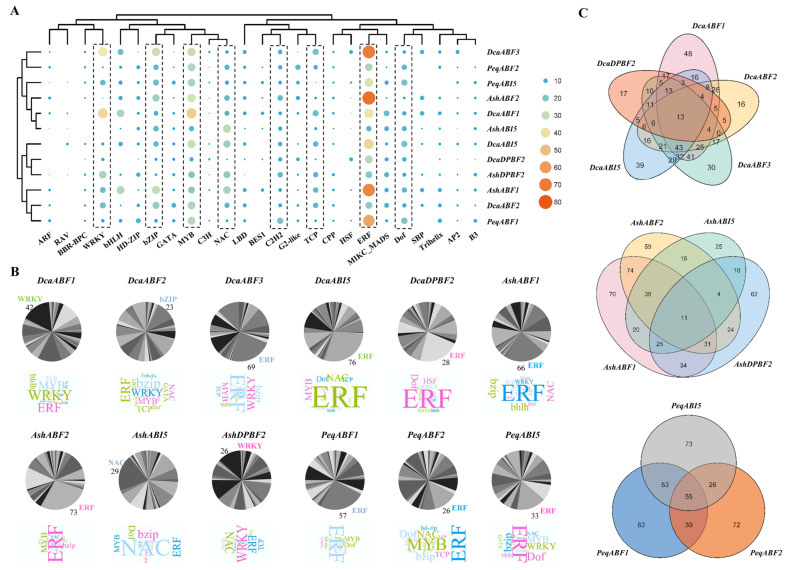
The putative TF regulatory network analysis of *AREB/ABF*s from orchid plants. (**A**) An overview of the cluster of enriched TF regulators for all *AREB/ABF* genes. Highly enriched TFs are indicated with dash boxes. (**B**) The distribution of TF regulators for individual *AREB/ABF* genes using pie charts and word clouds. The font size is positively correlated with the number of corresponding TF regulators. (**C**) Venn diagram showing the overlapping TF regulators among *AREB/ABF*s from three orchid genomes.

To explore the potential roles of *AREB/ABF*s in orchid plants, a CRE analysis of their promoter regions was constructed. The results showed that hundreds of TFs belonging to over 30 different TF families, including ERF, MYB, C2H2, ARF, WRKY, bZIP, NAC, LBD, MADS, Dof, etc., were predicted as potential regulators of the orchid *AREB/ABF*s (Figure S1). The enriched predicted TFs were ERF, WRKY, bZIP, MYB, NAC, and Dof for all the *AREB/ABF*s. Based on the prediction results, *DcaABF1* and *AshABF1* possessed the greatest number of regulators (302 TFs), followed by *DcaABI5* (265 TFs), *AshABF2* (258 TFs), and *PeqABF1* (229 TFs) (Table S1). Additionally, the top seven gene families that were predicted to regulate all the *AREB/ABF*s were proposed, which were ERF, WRKY, MYB, bZIP, C2H2, Dof, and NAC (Figure 5A). We also compared the regulators for all the *AREB/ABF*s and found the ERF family to be the most enriched one, except for *AshABI5*, which was predicted to be mostly targeted by the NAC (29 TFs) family members (Figure 5B). Additionally, we studied the common and specific TF regulators for *AREB/ABF*s in each orchid species. The results showed that 13 and 11 TFs are common regulators of the *AREB/ABF*s from *D. catenatum* and *A. shenzhenica*, respectively (Figure 5C). Unexpectedly, a total of 55 TFs were found as the common regulators of the *AREB/ABF*s from *P. equestris*. Individual *AREB/ABF*s were also found targeted by specific TFs. For instance, *DcaABI5* was predicted to be specifically targeted by 39 TFs among five *AREB/ABF*s from *D. catenatum*, and more detailed analysis showed that *DcaABI5* was probably regulated by dehydration-responsive element-binding protein 1C (DREB1C, AT4G25470) and DERB1D (AT5G51990), two proteins involved in cold acclimation and drought tolerance [50,51,52]. Overall, the predicted TF regulatory network of the orchid *AREB/ABF*s implied their potential roles in growth development, stress response, and network associations.

### 2.6. Distinct Expression Profiles of Orchid AREB/ABF Genes in Different Tissues

**Figure 6:**
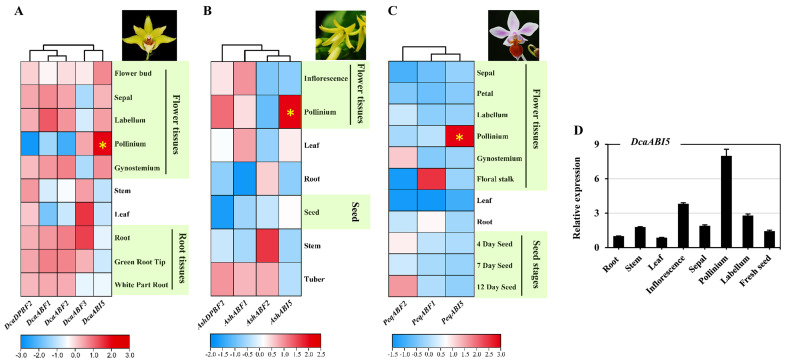
Expression pattern analysis for orchid *AREB/ABF* gene family. (**A**–**C**) Expression profiles of the *AREB/ABF* genes in different tissues/organs from indicated orchid species: *D. catenatum* (**A**), *A. shenzhenica* (**B**), and *P. equestris* (**C**). The heat map was constructed from the transcriptome data using TBtools-II with the log2-transformed RPKM values of each gene. The expression level was shown in color as the scale. (**D**) Expression patterns of *DcaABI5* in indicated tissues (specifically expressed in orchid pollinium highlighted with yellow asterisk). *DcaActin7* was used as the control. Three independent biological experiments, each with three technical replicates, were performed.

To explore the expression profiles of the orchid *AREB/ABF* genes, their transcript abundance patterns were analyzed using transcriptome data available in public database (http://orchidbase.itps.ncku.edu.tw/est/home2012.aspx, accessed on 2 October 2021). The results showed most of the *AREB/ABF* genes had tissue-specific expression patterns (Figure 6A–C). For instance, *DcaABF1* and *DcaABF2* were abundantly expressed in most of the tissues except for the leaves, stems, and pollinium. Instead, *DcaABF3* was highly expressed in the leaves, roots, and stem. *DcaDPBF2* was found present in all tissues except for the pollinium, while *DcaABI5* was mainly distributed in flower tissues, especially in the pollinium. In *A. shenzhenica*, *AshABF1* and *AshDPBF2* were mainly expressed in the flower tissues, leaves, and tubers, while *AshABF2* was highly presented in the stems. Notably, *AshABI5* was dominantly found in the pollinium. A similar expression pattern was found in *PeqABI5* from *P. equestris*, which was strongly accumulated in the pollinium.

Transcriptome analysis showed that *ABI5* in *Arabidopsis* was dominantly expressed in developing seeds (Figure S2). Since the expression pattern of *DcaABI5* in the seeds is not available in public databases, we performed RT-qPCR and found the comparative expression of *DcaABI5* in fresh seeds (Figure 6D). These findings revealed that *ABI5* from these orchid plants presented high expression in the pollinium but low expression in the seeds, suggesting the diverse role of ABI5 in orchid species.

### 2.7. Functional Analysis of DcaABI5

**Figure 7:**
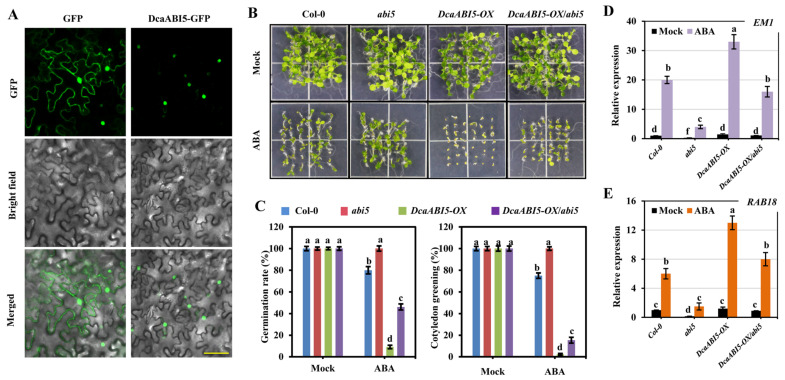
DcaABI5 is able to rescue *ABI5* mutation in *Arabidopsis*. (**A**) Confocal microscopy images for the subcellular localization of DcaABI5 in tobacco leaf. (**B**) Representative images for 7-day-old seedlings of Col-0, *abi5*, *DcaABI5* overexpression lines in WT (*DcaABI5*^OE^) and *abi5* (*DcaABI5*^OE^/*abi5*) germinated on half MS medium supplemented without or with 0.5 μM ABA. (**C**) Statistical analysis of seed germination and greening cotyledon percentages of the various genotypes in response to ABA. Seed germination was recorded after 3 days of stratification, and cotyledon greening was recorded 7 days after stratification on half MS medium supplemented without or with 0.5 μM ABA. Data indicate mean ± SD (*n* = 3) with at least 100 seeds for each replicate of each genotype. (**D**,**E**) *DcaABI5* regulated expression of stress-responsive genes. RT-qPCR analysis was performed to examine the relative transcript levels of *EM1* and *RAB18* in indicated plants treated without or with 10 μM ABA. *Actin2* was used as the control. Three independent biological experiments were carried out, each with three technical replicates. Bars with different letters indicate significant differences from the control as determined using one-way ANOVA, *p*-value < 0.05.

ABI5 as a typical bZIP protein is well known in response to ABA signaling [36]. The different expression profiles in *DcaABI5* compared with *ABI5* from *Arabidopsis* inspired us to investigate the role of ABI5 in *D. catenatum*. We firstly investigated the subcellular localization of DcaABI5-GFP using a fluorescence confocal microscope. The results showed that DcaABI5 was mainly presented in the nucleus of the tobacco leaf epidermal cells (Figure 7A). As a control, GFP was distributed throughout the cell.

To reveal the role of DcaABI5 in ABA signaling, we introduced the T-DNA insertion line *abi5* from *Arabidopsis* and overexpressed *DcaABI5* in this genetic background. The expression level of *DcaABI5* was confirmed using RT-qPCR in the transgenic plants, and individual lines with a higher level of *DcaABI5* were selected for further analysis (Figure S3). Further phenotypic analysis revealed that the wild-type Col-0 was sensitive to ABA with a reduced seed germination rate and less green cotyledons (Figure 7B,C). Notably, *DcaABI5* overexpression led to severe ABA sensitivity. On the contrary, *abi5* is insensitive to ABA, as previously reported [53], while the ABA-insensitive phenotype in *abi5* was largely rescued with *DcaABI5* overexpressed. We further examined the expression patterns for representative targets of ABI5, including *EM1* and *RAB18* [54]. The results showed that *DcaABI5* overexpression significantly promoted the expression of *EM1* and *RAB18* upon ABA treatment in *Arabidopsis* (Figure 7D,E). Additionally, *DcaABI5* overexpression also efficiently rescued the transcriptional regulation of these target genes in the *abi5* mutant.

Collectively, these data suggested that DcaABI5 also played an essential role in the ABA signaling pathway.

## 3. Discussion

Many orchid plants grown in wild forests usually suffer from environmental stresses, such as drought, low temperature, and light. It is thus important to learn the fundamental mechanisms behind the survival strategies in orchids. Several studies have proved the importance of ABA signaling to the plant stress response [55,56]. The *AREB/ABF* subgroup from the bZIP gene family has been demonstrated to play an indispensable role in the ABA signaling pathway for plants’ adaptation to external stresses [19,21,29]. Nevertheless, detailed information concerning the characteristics and functions of orchid AREB/ABFs, particularly their role in stress responses, largely remains unclear.

In our study, a total of twelve *AREB/ABF* genes were identified from three orchid species, and sequence analysis suggested that these AREB/ABFs contained all the conserved domains, such as C1–C4 and bZIP (Figure 1). This feature certainly provides full support for the identification of the AREB/ABF subgroup family in orchids. Compared to the numbers of the AREB/ABF family in *Arabidopsis* (nine) and rice (seven), the size of this gene family is smaller in orchid species, with five, four, and three in *D. catenatum*, *A. shenzhenica*, and *P. equestris*, respectively. It seems that the number of members of the orchid *AREB/ABF* gene family does not correlate well with their genome size. As compared to the genome size in *Arabidopsis* (125 Mb) and rice (480 Mb), the genomes of *D. catenatum* (1.11 Gb) and *P. equestris* (1.16 Gb) are substantially larger. Gene duplication events have been shown to play an important role in genome expansion for the production of large numbers in gene families [57]. Notably, whole-genome duplication events have been found to occur in all modern orchids, which may be related to their diversification [10,11,58,59]. For instance, the gene expansion in the SWEET gene family from *Dendrobium chrysotoxum* may be associated with enrichment in polysaccharides [59]. However, the number of *AREB/ABF* genes from the three orchid species is smaller than that from *Arabidopsis* and rice, suggesting that gene duplication in the orchid *AREB/ABF* genes might not have occurred. The MADX-box gene family in orchids is also smaller than that in *Arabidopsis* (107 genes) and rice (80 genes), as only 51 and 63 putative ones were identified in *P. equestris* and *D. catenatum*, respectively [9,10,60]. Despite having fewer MADS-box genes, orchids contain more ones related to floral organ production, suggesting that the higher diversity of MADS-box genes in orchids might be associated with specific floral morphological traits [60]. It is also possible that the genetic diversity of the *AREB/ABF* genes in orchids might be associated with specific biological functions. Interestingly, all the orchid *ABF* genes were found clustered into group A as a unique subgroup apart from those homologs from *Arabidopsis* and rice (Figure 2), implying that these genes probably share independent functions and functional redundancy in orchids. 

As observed in other plants [42,43,44,45,46,47,48], phylogenetically close *AREB/ABF*s members always have relatively consistent compositions of motifs and exons/introns, suggesting that these AREB/ABF subgroup proteins might share a similar functionality (Figure 3). Of the 10 conserved motifs identified in all the AREB/ABF proteins, motif 1 and motif 2-5 were highly consistent, which consist of the conserved bZIP domain and the phosphorylation domains C1–C3, respectively (Figure 3). The bZIP domain is the most representative characteristic and critical to the function of these transcription factors. The C1–C4 domains are supposed to play a key functional role in decoding different signals. For instance, the domains C1–C3 were phosphorylated by SnRK2/SnRK3 under hyperosmotic cold stress in [61]. Recent studies have also demonstrated that the novel osmosensor DROOPY LEAF1 (DPY1), a leucine-rich repeat receptor-like kinase that is localized to the cell surface, mediates SnRK2 activation and global downstream phosphorylation events against drought stress [62,63]. It reinforced the core role of AREB/ABFs in signal integration in the complex stress-signaling network. In addition to SnRKs, calcium-dependent protein kinases (CDPKs) also mediate the phosphorylation of the ABFs in the C1–C4 domains for stress tolerance, such as cold [64]. Apart from the C1–C3 domains, the C4 domain from AREB/ABF proteins is essential to their protein stability, as the deletion of the C4 domain accelerates the degradation of *Arabidopsis* AtABF1 and AtABF3 in vitro [65]. We found that motif 7 is uniquely presented in the C4 domain in ABF proteins. It is likely that a 14-3-3 interaction site in motif 7 contributes to the stability of ABF genes [65,66], indicating the genetic diversity of the AREB/ABF family.

*Cis*-elements play crucial roles in the regulation of gene transcription. Here, many regulatory *cis*-elements related to hormone response, stress response, and growth development were predicted in the promoters of the orchid *AREB/ABF* genes (Figure 4). Phytohormones are important to plant development and responses to various environmental stimulus [67]. We found that *cis*-elements related to ABA, JA, and ethylene are mostly enriched, suggesting that the expression of *ABRE/ABF* genes in orchids may be closely related to these variable phytohormones, underlying different environmental changes. There is no doubt that all the *AREB/ABF*s underling ABA regulation have ABRE elements. This is consistent with the other AREB/ABF genes reported [29,48,49]. Hence, AREB/ABF genes certainly contribute to drought stress tolerance in orchids. JA is also responsible for the regulation of important growth and developmental processes and responses to environmental stresses, such as stomatal opening, external damage, etc. [68,69]. Orchid AREB/ABF genes probably function in the cross-talk between the two hormones in mediating stomatal movement in response to dehydration and rehydration or invader/pathogen attacks on attractive flowers. Unexpectedly, numerous *cis*-elements related to ethylene response were predicted in orchid *AREB/ABF* genes (Figure 4), which are different to those reported in other plant species, including tomato, mei (plum), and jute [40,45,48]. Consistently, the predicted potential TFs binding to the *AREB/ABF*s revealed that ethylene response factors (ERFs) are the most abundant (Figure 5B). It has been reported that ethylene and ABA jointly mediate seed germination, root growth, and fruit ripening [70,71,72]. ERF55 and ERF58 in *Arabidopsis* were found to directly regulate the transcription of *ABI5* for seed germination [73]. Thus, ethylene probably plays a role in seed germination through the regulation of *AREB/ABF*s in orchids, which is extremely complex and largely remains to be illustrated. Additionally, stress-responsive elements related to drought, cold, and wound stresses were evenly predicted in the promoters of the *AREB/ABF* genes. Notably, *cis*-elements related to light responsive were highly redundant. Overall, the results suggested a close relationship between the expression of *AREB/ABF*s and the growth of orchids under environmental stress conditions.

To better explore the function of orchid *AREB/ABF*s, their expression patterns were evaluated using publicly available transcriptome data. As these data were generated for different orchid species, the number and identity of the tissues or developmental stages analyzed were not identical. Though differences in the expression profiles of these *AREB/ABF*s from different orchid species were presented, some commonalities were found. For instance, the *ABI5* genes from three orchid species were found dominantly expressed in the pollinium (Figure 6A–C), indicating that the orchid ABI5 gene probably plays an important and diverse role in pollen development or fertilization. Unexpectedly, the expression of the *ABI5* genes was relative low in orchid seeds, whereas *ABI5* from *Arabidopsis* is mostly expressed in the seeds for dormancy and germination [53,54]. Since ABI5 was conserved, we found that the overexpression of DcaABI5 conferred increased the ABA sensitivity during seed germination and cotyledon greening, as expected (Figure 7B). In addition, the ABA-insensitive phenotype of the *abi5* mutant from *Arabidopsis* could be significantly rescued with DcaABI5 overexpressed, suggesting the conserved role of DcaABI5 in the ABA signaling pathway.

## 4. Materials and Methods

### 4.1. Identification of the AREB/ABF Genes in the Orchid Species

To identify the genes encoding the AREB/ABFs in orchids, all nine *Arabidopsis* AREB/ABFs were used as queries to search for orchid genomes in OrchidBase 4.0 (http://orchidbase.itps.ncku.edu.tw/est/home2012.aspx, accessed on 3 July 2021) for candidate AREB/ABF sequences using the BLASTP program. All the candidate protein sequences were further analyzed using SMART (http://smart.embl-heidelberg.de/, accessed on 8 July 2021) to confirm the integrity of the C1-C4 and bZIP domains. All non-redundant and confident genes were finally gathered and assigned as the orchid *AREB/ABF* genes. The features, including isoelectric point (pI) and molecular weight (MW), of the AREB/ABFs were analyzed using ExPASy-ProtParam (Expasy 3.0; http://web.expasy.org/protparam/, accessed on 10 July 2021).

### 4.2. Analyses of Conserved Motifs, Exon–Intron Structures, and Cis-Regulatory Elements in Orchid AREB/ABF Genes

The exon–intron structures of all the orchid *AREB/ABF* genes were analyzed using the Gene Structure Display Server (GSDS) program [74]. The conserved motifs in the AREB/ABF proteins were searched using Multiple Em for Motif Elicitation (MEME) v5.5.5) (http://meme-suite.org/tools/meme, accessed on 15 July 2021) and were further analyzed using the InterPro database (https://www.ebi.ac.uk/interpro/, accessed on 18 July 2021). To identify potential *cis*-elements in the *AREB/ABF* genes, 2000 bp sequences upstream of their translation initiation site ATG were first obtained using TBtools-II [75] and then analyzed using the PlantCARE database (http://bioinformatics.psb.ugent.be/webtools/plantcare/html/, accessed on 12 September 2021).

### 4.3. TF Regulatory Network Analysis

The Plant Transcriptional Regulatory Map (PTRM) (http://plantregmap.gao-lab.org/, accessed on 15 April 2022) was used to predict potential regulatory interactions between TFs in the upstream (2000bp) regions of the orchid *AREB/ABF*s with a threshold (*p*-value ≤ 1 × 10^−7^). *Arabidopsis* was the selected plant species. The heat map, word clouds, and Venn diagrams were constructed using TBtools-II [75].

### 4.4. Expression Profiles of AREB/ABF Genes in Different Tissues

The expression levels of *AREB/ABF* genes from *D. catenatum*, *P. equestris*, and *A. shenzhenica* were estimated according to the published RNA sequencing data OrchidBase 4.0 [76,77]. The expression levels of *AREB/ABF* genes from *Arabidopsis* were derived from The Bio-Analytic Resource for Plant Biology (https://www.bar.utoronto.ca/, accessed on 20 September 2021). The expression heat map and cluster analyses were constructed using TBtools-II [75].

### 4.5. Plant Materials and Treatments

The wild-type *Arabidopsis thaliana* Col-0 accession was used in this study. And the mutant used in this study was *abi5* (SALK_013163), as previously reported [78]. The plant growth was carried out in a culture room at 22 °C over a long-day photoperiod (16 h:8 h; light:dark, respectively) with a photon flux density of 180 mmol photons m^−2^ s^−1^ and an ambient humidity of 70%. For assessment of the phenotypes, seeds from each genotype were sterilized with 75% ethanol for 10 min and washed more than five times with sterile water. After that, the sterilized seeds were sown on half MS medium plates with 1% (*w*/*v*) sucrose and 1.5% (*w*/*v*) agar, pH 5.8, or half MS medium plates supplemented without or with 0.5 μM ABA and left at 4 °C for 2 days before being transferred into a growth chamber. The seed germination (emergence of radicles) was scored and photographed after 3 days of stratification, and the cotyledon greening was recorded 7 days after stratification.

### 4.6. Vectors Construction and Plant Transformation

To investigate the subcellular localization of DcaABI5, the coding region of *DcaABI5* was cloned into the modified gateway-compatible binary vector pGWB414. The binary vector was firstly confirmed using sequencing and then introduced into *Agrobacterium tumefaciens* (*A. tumefaciens*) strain GV3101 cells. For transient transformation, the transformed *A. tumefaciens* cells containing each construct were prepared to an OD600 of 0.8 and then injected into *N. benthamiana* leaves. The transient expression of the fusion protein was examined using a confocal laser-scanning microscope (Leica SP8; Leica Microsystems GmbH, Solms, Germany) at 48 h after transformation. Transgenic *Arabidopsis* plants were generated using the *Agrobacterium tumefaciens*-mediated floral dip method [79]. All the transgenic lines used in this study were homozygous T3 lines. The primers for the vector construction are listed in Table S2.

### 4.7. Analysis of the Gene Expression with RT-qPCR Analysis

The total RNA extraction from the indicated tissues and organs, first-strand cDNA, and RT-qPCR assay were performed following our previous study [80]. The transcript data were calculated using 2^−ΔΔCt^ to quantify the gene expression levels [81]. *Actin2* from *Arabidopsis* and *DcaActin7* from *D. catenatum* were used as the internal controls. Each experiment was performed with three replicates. The primers for the RT-qPCR are listed in Table S2.

### 4.8. Statistical Analysis

The data from the seed germination and cotyledon greening ratio were processed using GraphPad Prism 8.00 software. The statistical details of the experiments can be found in the corresponding figure legends. The statistical analysis was performed using one-way ANOVA (Tukey’s multiple-comparisons test) in GraphPad.

## 5. Conclusions

*AREB/ABF* genes are essential to ABA signaling pathways for plant growth and adaptation to environmental stresses. Nevertheless, no report on the *AREB/ABF*s from orchids has previously been presented. In this study, twelve *AREB/ABF* genes were identified within three orchids’ genomes (*D. catenatum*, *A. shenzhenic,* and *P. equestris*) and classified into three groups via a phylogenetic analysis. The *cis*-element analysis suggested that *AREB/ABF*s might be widely involved in various phytohormone responses, including ABA, JA, and ethylene. The orchid AREB/ABF-mediated regulatory network constructed through *cis*-regulatory element (CRE) analysis revealed that the ethylene response factor (ERF) gene family was the most abundant as a potential regulator. Expression profile analysis based on public transcriptomic data showed that most of the *AREB/ABF* genes have distinct tissue-specific expression patterns in orchid plants. Notably, the representative *AREB/ABF* member *ABI5* from orchid species was found specifically expressed in the pollinium. Additionally, overexpression of ABI5 from *D. catenatum* conferred ABA sensitivity and rescued the ABA-deficient mutant *abi5* in *Arabidopsis*. Taken together, our results will provide valuable information on *AREB/ABF* genes and a perspective for further functional characterization of potentially important ones in orchids.

## Figures and Tables

**Table 1:** Characteristics of *AREB/ABF* subfamily members in three orchid species.

Species	Gene	Gene Id	Coding Sequence (CDS) Length	Protein Length (aa)	Molecular Mass (kDa)	Theoretical pI	Sub-Cellular Location
*Dendrobium catenatum*	*DcaABF1*	Dca012913	1224	407	43.50	7.7	Nucleus
	*DcaABF2*	Dca011277	1221	406	42.69	9.13	Nucleus
	*DcaABF3*	Dca006042	1191	396	42.67	9.19	Nucleus
	*DcaABI5*	Dca002027	1074	357	38.95	7.60	Nucleus
	*DcaDPBF2*	Dca016354	936	311	34.83	9.50	Nucleus
*Apostasia shenzhenica*	*AshABF1*	Ash014915	1143	380	40.72	8.71	Nucleus
	*AshABF2*	Ash016767	1278	425	45.56.	9.00	Nucleus
	*AshABI5*	Ash004480	1188	395	42.51	5.47	Nucleus
	*AshDPBF2*	Ash013161	1023	340	37.18	8.74	Nucleus
*Phalaenopsis equestris*	*PeqABF1*	Peq004088	1221	406	42.60	9.39	Nucleus
	*PeqABF2*	Peq011139	1191	396	42.57.	8.65	Nucleus
	*PeqABI5*	Peq013682	1068	355	39.91	9.42	Nucleus

## Data Availability

Data are contained within the article and Supplementary Materials.

## Supplementary Materials

The following supporting information can be downloaded at https://www.mdpi.com/article/10.3390/plants13060774/s1. Figure S1: The statistics of predicted TFs binding to *AREB/ABF*s in orchids. Figure S2: Expression pattern analysis for *AREB/ABF*s from *Arabidopsis*. The heat map was constructed from the transcriptome data using TBtools-II with the log2-transformed RPKM values of each gene. The expression level was shown in color as the scale. Figure S3: Analysis of the *DcaABI5* expression in individual transgenic plants. RT-qPCR analysis was performed to examine the relative transcript levels of *DcaABI5. Actin2* was used as the control. Three independent biological experiments were carried out, each with three technical replicates. Table S1: List of TFs binding to *AREB/ABF*s in orchids. Table S2: List of the primers used in this study.
