# Supplementary figures and images for: Identification and Characterization of the AREB/ABF Gene Family in Three Orchid Species and Functional Analysis of DcaABI5 in Arabidopsis
Source: Plants (Basel). 2024 Mar 8;13(6):774. doi: 10.3390/plants13060774 (PMC10974128; doi:10.3390/plants13060774)

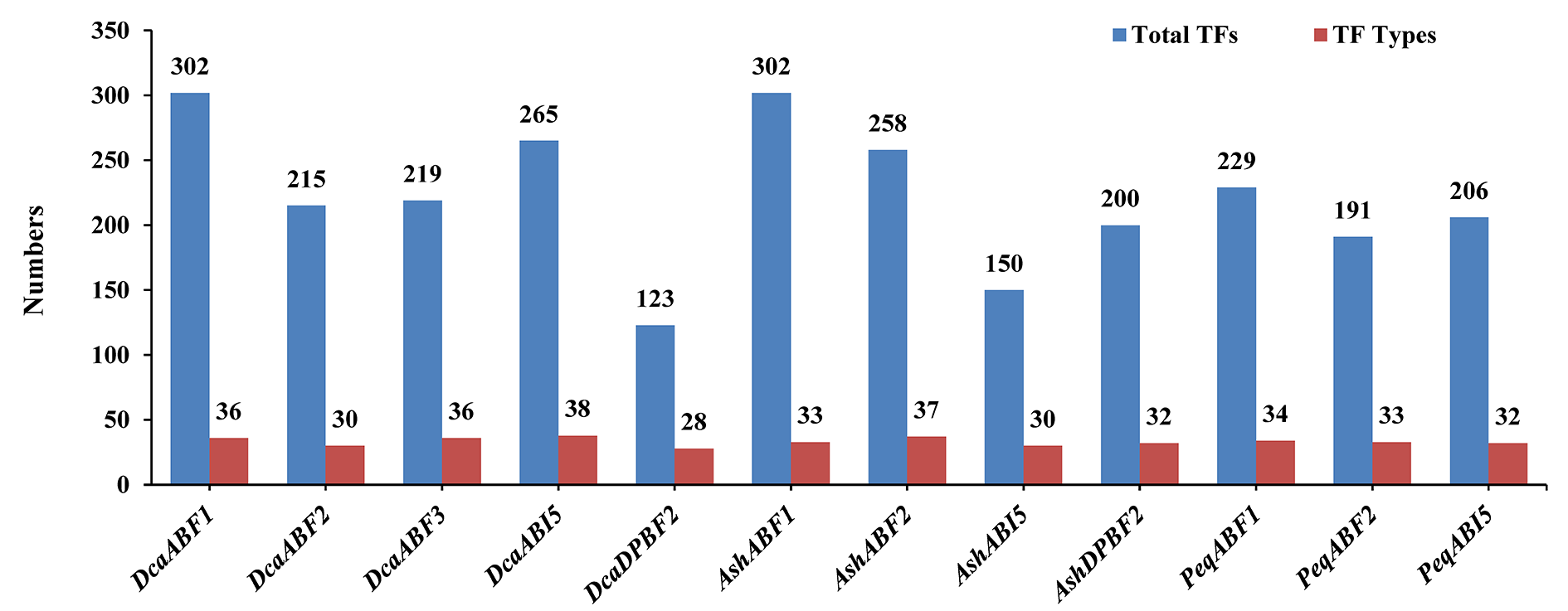

Supplement: Supplementary file 1 [file plants-13-00774-s001.zip › Figure S1.tiff]

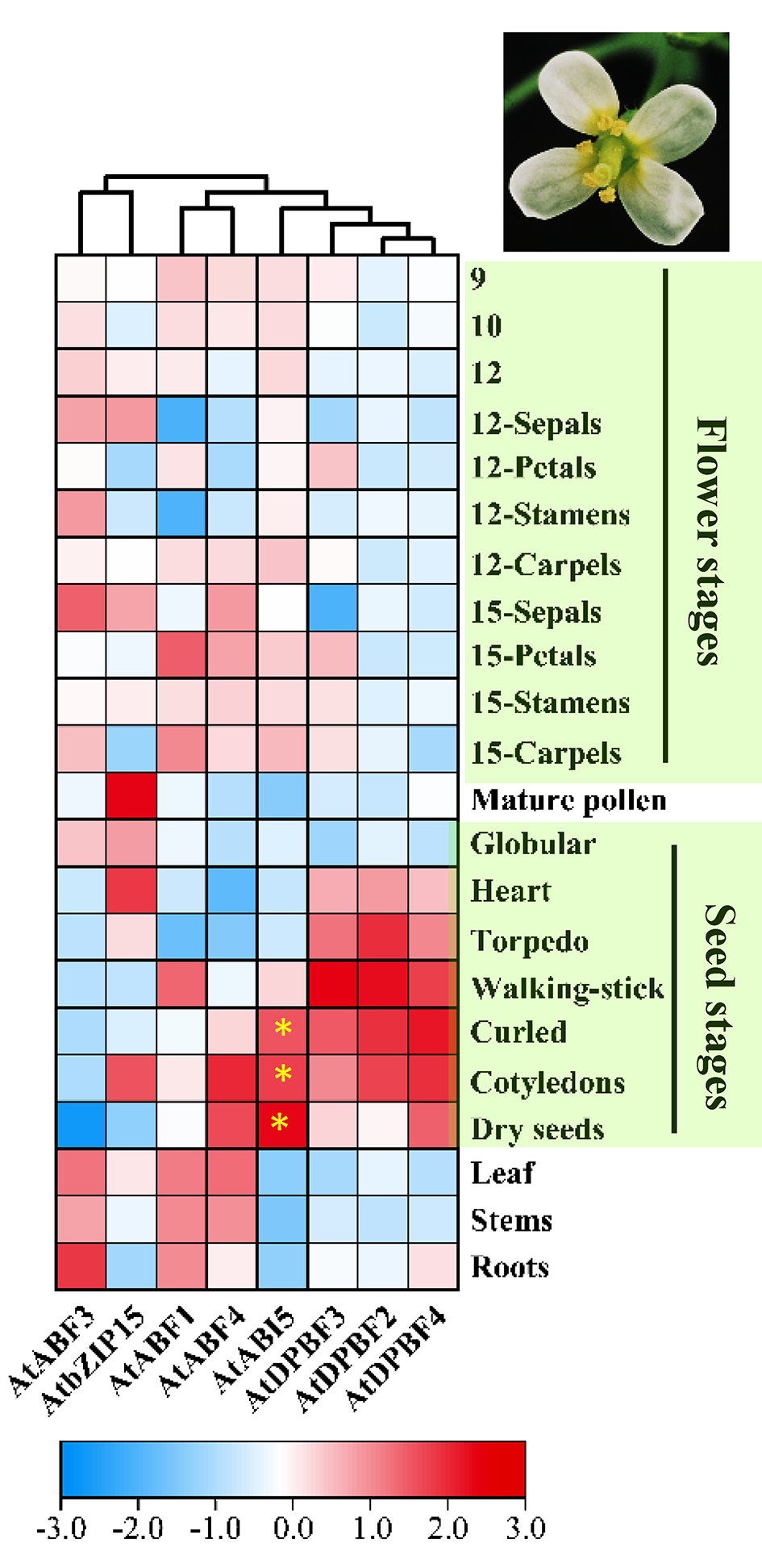

Supplement: Supplementary file 1 [file plants-13-00774-s001.zip › Figure S2.tiff]

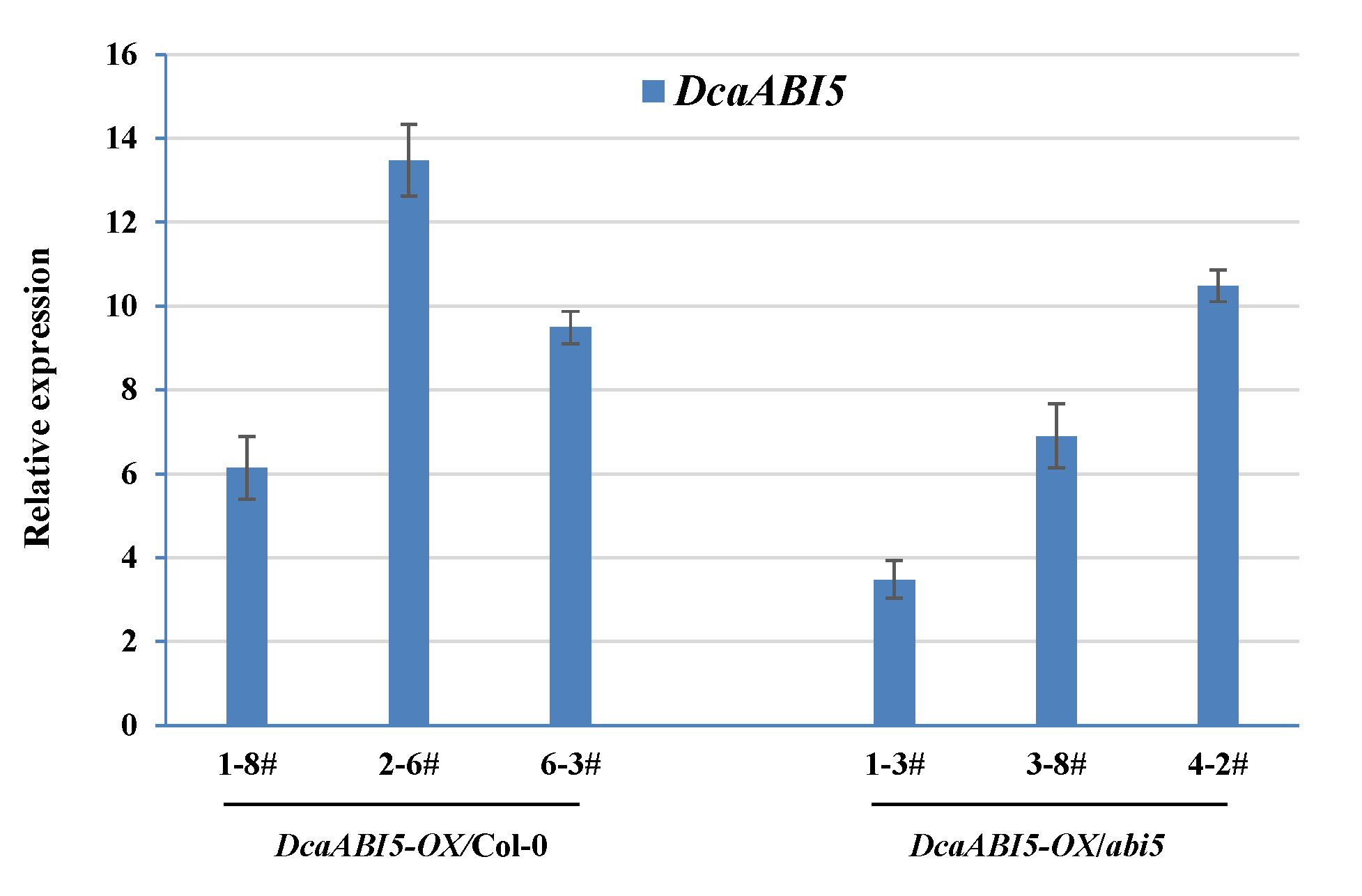

Supplement: Supplementary file 1 [file plants-13-00774-s001.zip › Figure S3.tiff]
